# Research Progress of Imaging Methods for Detection of Microvascular Angina Pectoris in Diabetic Patients

**DOI:** 10.3389/fcvm.2021.713971

**Published:** 2021-09-21

**Authors:** Yiming Qi, Lihua Li, Guoquan Feng, Chen Shao, Yue Cai, Zhongqun Wang

**Affiliations:** ^1^Department of Cardiology, Affiliated Hospital of Jiangsu University, Zhenjiang, China; ^2^Department of Pathology, Affiliated Hospital of Jiangsu University, Zhenjiang, China; ^3^Department of Radiology, Affiliated Hospital of Jiangsu University, Zhenjiang, China

**Keywords:** microvascular angina, diabetes, diagnosis, imaging methods, research progress

## Abstract

Diabetes is a complex metabolic disease characterized by hyperglycemia. Its complications are various, often involving the heart, brain, kidney, and other essential organs. At present, the number of diabetic patients in the world is growing day by day. The cardiovascular disease caused by diabetes has dramatically affected the quality of life of diabetic patients. It is the leading cause of death of diabetic patients. Diabetic patients often suffer from microvascular angina pectoris without obstructive coronary artery disease. Still, there are typical ECG ischemia and angina pectoris, that is, chest pain and dyspnea under exercise. Unlike obstructive coronary diseases, nitrate does not affect chest pain caused by coronary microvascular angina in most cases. With the increasing emphasis on diabetic microvascular angina, the need for accurate diagnosis of the disease is also increasing. We can use SPECT, PET, CMR, MCE, and other methods to evaluate coronary microvascular function. SPECT is commonly used in clinical practice, and PET is considered the gold standard for non-invasive detection of myocardial blood flow. This article mainly introduces the research progress of these imaging methods in detecting microvascular angina in diabetic patients.

## Introduction

Diabetes is a complex metabolic disease characterized by hyperglycemia (1). It affects all organs of the body, and there are many kinds of complications. According to the World Health Organization, there are more than 100 kinds of complications in patients with diabetes, including vascular and non-vascular complications. Blood vessel complications can be divided into macrovascular and microvascular complications, and many diabetic patients have vascular complications in the early diagnosis of diabetes (2). Diabetic microvascular angina is a kind of microvascular disease caused by diabetes. Kemp (3) first described this syndrome with typical angina pectoris symptoms and myocardial ischemia, but with normal coronary angiography as “cardiac X syndrome.” Until 1985, Cannon called this disease coronary microvascular angina (CMVA). Although people have a deep understanding of the obstructive coronary disease, they lack understanding of non-obstructive angina caused by coronary microvascular dysfunction (CMVD). von Scholten et al. (4) showed that the incidence of CMVD in patients with type 2 diabetes was significantly higher than that in people without diabetes. Diabetic patients have a higher risk of developing obstructive coronary artery disease (5), but coronary CMVD should not be ignored (6). The latter can lead to coronary microvascular angina pectoris, resulting in angina symptoms. Still, coronary angiography shows no apparent stenosis. The severity of this disease in women is higher than that in men, this may be related to female unique aldosterone hyperresponsiveness (7). CFR is a recognized index for evaluating CMVD. In addition, we can also use the index of microcirculatory resistance (IMR) to evaluate CMVD (8). More than 490,000 invasive coronary angiography (ICA) operations were performed in Japan, according to a 2019 JROAD report. However, the number of percutaneous coronary interventions was only 271,000 (9). This demonstrates that many invasive examinations are unnecessary, many patients do not have severe obstructive coronary disease. Therefore, we need to pay more attention to non-invasive ways. At present, the standard imaging diagnosis methods are SPECT, PET, CMR, and MCE. Accurate diagnosis of diabetic microvascular angina is of great help to the treatment and prognosis of patients.

## Diabetic Microvascular Dysfunction

Coronary microcirculation determines 95% of the myocardial blood flow resistance, while the epicardial coronary artery only accounts for 5% (10). So microcirculation plays a significant role in regulating myocardial blood supply. Coronary microcirculation is composed of anterior arterioles, arterioles, and capillaries. It regulates capillaries' blood flow through specific mechanisms to meet the dynamic demand of myocardial blood supply (11), including the endothelium-dependent mechanism and endothelium-independent mechanism. When the body is suffering from diabetes, coronary microcirculation will be affected by a series of factors such as hyperglycemia, inflammatory reaction, cardiac autonomic nerve dysfunction, etc. It leads to myocardial ischemia, eventually CMVA.

Studies have shown that this injury is similar in patients with type 1 and type 2 diabetes, and chronic hyperglycemia is a crucial factor leading to CMVD (12). The main feature of CMVD in diabetic patients is endothelial dysfunction, which leads to the impairment of endothelial-dependent mechanisms. In some cases, endothelial-independent mechanisms may also be affected as well (13). In people with diabetes, microcirculation first occurs functional changes, then structural changes (14). These functional changes include endothelial dysfunction, autonomic nerve dysfunction, etc. Structural changes include thickening of the arterial wall, capillary basement membrane, formation of deposits in the small artery wall, and small coronary artery wall fibrosis. Recently, some scholars suggested that (15) endothelial dysfunction is related to the down-regulation of MCT4. Hyperglycemia and inflammatory factors can downregulate the expression of MCT4. This results in lactic acid transport disorder and intracellular pH imbalance, eventually leading to endothelial cell apoptosis and endothelial dysfunction.

The incidence of CMVA is high, but because there is no imaging performance of obstructive coronary disease, it is easy to cause misdiagnosis and missed diagnosis, which has seriously affected people's lives. The COVADIS conducted multinational, multiracial, and multicenter prospective observational studies in 14 research centers in 4 continents, 7 countries to explore the clinical characteristics and prognosis of patients with MVA and the impact of gender and race on patients CMVA (16). From June 2015 to December 2018, the study prospectively included 688 MVA patients, with all patients not receiving trial treatment during the observation phase. The results showed that MVA patients had a high risk of MACE regardless of gender and race, with an average MACE rate of 7.7% years. However, Caucasian whites were significantly higher than Asian populations and were associated with hypertension and previous coronary heart disease, with poor quality of life in women compared to men.

To better develop treatment strategies, CMVD can be divided into four different types. Ca Mici and Filippo (17) divided coronary microvascular dysfunction into four types: type I: CMVD without myocardial disease and coronary stenosis; type II: CMVD caused by myocardial disease; type III: coronary artery occlusion combined with CMVD; type IV: iatrogenic CMVD. In patients with diabetes, due to some unique pathophysiological mechanisms, Type I and type III CMVD are most likely to occur, and type I CMVD is the earliest in patients with diabetes (13).

## Diagnosis of CMVA

At present, the diagnostic criteria of CMVA are proposed by COVADIS, including the following four items: (1) The presence of symptoms indicating myocardial ischemia; (2) There was objective evidence of myocardial ischemia; (3) No obstructive coronary disease; (4) Evidence of coronary microvascular dysfunction. When the four conditions are met, coronary microvascular angina can be diagnosed; when there are standards 1 and 3, but standards 2 and 4 have only one item, It can only be diagnosed as suspected coronary microvascular angina (18).

Because the current technology does not allow us to observe coronary microcirculation directly (19), we can only indirectly evaluate the microcirculation function through some methods to obtain evidence of myocardial ischemia. Myocardial ischemia can be caused by epicardial coronary artery occlusion or CMVD. When both of them have dysfunction, it can also cause myocardial ischemia (20).

Coronary flow reserve (CFR) is a recognized index to evaluate coronary blood flow. Gould KL first proposed CFR in 1974. It refers to the ratio of mean blood flow in the congestive phase to mean blood flow at rest after the application of vasodilator. The normal value of CFR is between 2 and 2.5 (21). The coronary microvascular disease can cause a decrease in CFR (≤ 2). CFR is determined by both large and microvessels of the coronary artery. Therefore, the reduction is caused by CMVD when CAD is excluded. Some studies have shown that the causes of CFR decrease in patients with diabetes are different in different periods. In patients with early diabetes, the reduction of CFR is mainly caused by increased baseline blood flow velocity. In contrast, the decrease of CFR in patients with advanced diabetes mellitus is mainly caused by decreased blood flow velocity during hyperemia (22). It is crucial to determine CFR in diabetic patients. Even in patients with diabetes without known CAD, their Cardiac Mortality is comparable to non-diabetic patients with known CAD if CFR is impaired (23). At present, CFR can be measured by both invasive and non-invasive methods. The former is mainly coronary Doppler flow guide wire, while the latter includes SPECT, CMR, and PET explicitly. MCE has also been proved to be able to evaluate coronary microcirculation (24).

TIMI myocardial imaging grading can also evaluate coronary microcirculation. Its principle is to analyze the time required for contrast agents to enter myocardial tissue, divided into 0–3 grades (25). TIMI can be used as a semi-quantitative index to reflect the perfusion state of coronary microcirculation. However, TIMI grading is limited by observer variability. Studies have shown that (26) when measured by two independent laboratories, the coincidence rate of TIMI assessment is only 83%. When the results of the third laboratory exist, the coincidence rate further decreases. When the TFC of the left anterior descending artery was modified with 1.7, we can obtain corrected TIMI classification (CTFC). Compared with TIMI flow classification, CTFC is more objective and repeatable. It can evaluate microvascular dysfunction in patients with microvascular angina pectoris. Ding et al. (27) proposed a new index TMP frame counting (TMP-FC) based on TIMI, myocardial angiography was quantified by frame counting. Ge et al. (28) suggested that TMP-FC ≥95.5 was an independent predictor of MVD (OR = 11.61, *P* < 0.001). Moreover, the sensitivity and negative predictive value of TMP-FC in the diagnosis of CMVD were significantly improved. TMP-FC was directly proportional to the degree of CMVD progression, so that it can present disease progression.

## Research Progress of Imaging Methods for Detection of Microvascular Angina pectoris

### Single-Photon Emission Computed Tomography

SPECT usually uses thallium-201 (^201^Tl) or technetium-99m (^99m^Tc) labeled tracers (29) to record the radioactivity of the myocardium under resting and loading conditions. According to the phenomena of myocardial perfusion decrease and defect in the two states, we can diagnose CMVD after eliminating the epicardial coronary artery stenosis. The advantage of SPECT in diagnosing CMVD is its higher sensitivity. Compared with PET, SPECT is cheaper (30), so it is more frequently-used in the clinic.

In recent years, solid-state detector technology based on cadmium-zinc telluride (CZT) has become a hot spot in SPECT development. Recently, the CZT-SPCET has achieved a commodified supply. Two cardiac-centered CZT cameras are available commercially-Discovery NM530c and D-SPECT. CZT-SPECT has gradually expanded the scope of clinical applications due to the technical advantages. The traditional anger camera is limited by low photon sensitivity and low inherent spatial resolution and needs a high radioisotope dose to ensure image quality. CZT-SPECT uses a semiconductor solid-state detector, directly converting gamma rays into electrical signals at room temperature (31). CZT crystal has higher photon sensitivity and higher energy resolution, so using a lower dose of radioactive tracer can also ensure image quality (32). The research of Duvall et al. (33) shows that the new single-photon emission tomography CZT camera technology can significantly reduce the radiation exposure and acquisition time on the premise of ensuring the imaging quality. CZT-SPECT can also lessen the injection dose of examiners. Nakazato et al. (34) applied CZT detector SPECT-MPI to 79 patients with the guarantee of image quality. When the acquisition time of the experimental group was 14 min, the average injection dose of the experimental group could be reduced to 92.5 MBq (2.5mCi). This is beneficial for protecting patients' health and improving the working efficiency of hospitals.

Due to the technical limitations, the standard semi-quantitative evaluation of myocardial perfusion cannot assess subtle changes in myocardial blood flow (MBF) regulation. The MPR obtained by CZT-SPECT can help to overcome the limitations of semi-quantitative MPI. CZT detector allows dynamic acquisition and quantification of MBF, Konstantin et al. (35) compared the ability of invasive coronary angiography and dynamic CZT-SPECT to assess myocardial blood flow. They found that CFR obtained by dynamic CZT-SPECT is consistent with CFR obtained by the invasive method. Acampa et al. (36) compared the ability of CZT-SPECT and PET imaging to evaluate myocardial absolute blood flow and blood flow reserve. It is found that there is a significant correlation between the latter and positron emission tomography. Therefore, CZT-SPECT can provide a more accurate myocardial blood flow. Because it can adopt a semi-automatic method, CZT-SPECT reduces the errors caused by operators and improves the accuracy and repeatability of diagnosis (37).

Interestingly, the advent of the CZT camera made the Simultaneous dual isotope ^201^Tl/^99m^Tc myocardial perfusion imaging possible. ^201^Tl is more linearly related to myocardial blood flow leading to an accurate identification of mild-ischemia. ^99m^Tc labeled tracers have nearly ideal physical imaging properties for gamma cameras, so it has a better imaging quality (38). However, it was found in Bernard Songy's studies that defect size in 20% of patients would be underestimated. This is mainly due to the lack of good scattering correction methods (39). But this at least proves that the goal is not out of reach. Moreover, with the appearance of the CZT camera, it is possible to reuse an old tracer, ^99m^Tc-Teboroxime, which has a high extraction rate in a wide range of coronary blood flow rates and is seldom used because of its short half-life. However, it may show unique advantages in clinics with the progress of technology (40).

The image reconstruction of dynamic SPECT is complicated. Because the camera rotates slowly, the dynamic data must be reconstructed from the projection, and the attenuation should be corrected appropriately. Therefore, Debasis Mitra developed a Spline Initialized Factor Analysis of Dynamic Structures (SIFADS) algorithm (41), which performed well in animal and human experiments and significantly reduced image noise and improved image quality. In addition, deep learning technology is developing rapidly and shows excellent potential in image acquisition and processing, which may be widely used in the future (42).

### Positron Emission Computed Tomography

PET is considered the gold standard for the non-invasive detection of myocardial blood flow (43). PET myocardial perfusion imaging (MPI) is a powerful tool for diagnosing coronary artery disease (44), which is widely used to detect cardiovascular function. Compared with SPECT-MPI, PET-MPI has higher sensitivity and specificity, can quantify myocardial blood flow more accurately (45), and provides higher quality images (46). Nevertheless, at the same time, it also has a higher inspection cost. The basic principle of PET-MPI is to dynamically analyze the signal of radionuclide tracer injected into a human body. If microcirculation disturbance occurs, it will show the perfusion defect at the corresponding position. At present, the commonly used tracers are mainly ^15^O-H_2_O, ^13^N-NH_3_, and ^82^Rb-RbCl. CFR can be obtained by detecting tracer signals at rest and under stress, respectively. If CFR decreases after eliminating obstructive coronary disease, CMVD is indicated.

Wanli et al. (47) evaluated the value of ^13^N-NH_3_ PET-MPI in the diagnosis of microvascular diseases. They found that the MBF of diabetic patients was lower than that of non-diabetic patients [(2.63 ± 0.98) and (3.62 ± 1.28) ml·min-1·g-1; *t* = −2.758, *P* = 0.008], and diabetes may be a risk factor for the decrease of MBF (OR = 0.254, 95% CI: 0.073–0.887), so diabetes is closely related to the occurrence of coronary microvascular disease. Studies have shown that PET-derived MBF and CFR are powerful tools for predicting future major adverse cardiac events. For example, a CFR decrease (<1.6) on 13N-ammonia PET and 82rubidium PET is an independent factor to predict MACE and early revascularization (48). We know that CFR in diabetic patients is closely related to cardiac death. PET is the gold standard for quantitative myocardial blood flow, so the use of PET is of great clinical significance for the accurate quantification of myocardial blood flow in diabetic patients.

It should be noted that the half-life of tracers used at present is very short, which requires hospitals to have cyclotrons to make tracers, which further increases the detection cost and has higher requirements for hospitals (49). PET tracer should have the characteristics of high specificity to molecular targets, high metabolic stability, high affinity to target tissues, low cost, and good safety (50). The new generation tracer ^18^F-flurpiridaz has a long half-life (110 min), low cost, and good imaging quality. Although it is still in the clinical trial stage, it has shown excellent performance (51). Compared with other PET radionuclides, ^18^F has the shortest positron range in tissues (52). It improves spatial resolution and may be widely used in microvascular imaging in the future. ^18^F-labeled ^18^F-FDG is a radiolabeled glucose analog. As with glucose, ^18^F-FDG passes into the cells through a glucose transport mechanism. However, ^18^F-FDG cannot participate in glycolysis and cannot be metabolized and excreted from cells. Ischemic sites can be imaging by using differences in ^18^F-FDG absorption in normal and ischemic myocardium (53). Because the glucose metabolism of cardiac muscle cells varies greatly in different physiological situations, strict control of the metabolic environment is required to accurately diagnose myocardial ischemia (54). In any case, the application of ^18^F-FDG contributes to the diagnosis of microvascular angina.

The unique feature of detecting microcirculation using PET is that PET obtains information on the molecular level (55). It can reflect the degree of damage to myocardial cells. For instance, [^18^F]FEDAC is commonly used in non-invasive visualization of neuroinflammation, inflammation of the lung, acute liver injury, and liver fibrosis (56), but recent studies have found that [^18^F] FEDAC also has good results in assessing the myocardial ischemic injury. Myocardial ischemia leads to myocardial cell mitochondrial dysfunction, then downregulation of translocator protein TSPO expression. Luo et al. (57) synthesized an ^18^F-labeled PET probe, N-benzyl-N-methyl2-[7,8-dihydro-7-(2-[^18^F]fluoroethyl)-8-oxo-2-phenyl-9Hpurin-9-yl]acetamide ([^18^F]FEDAC), with a high binding affinity for TSPO. They injected FEDAC into two groups of mice and analyzed the differences in TSPO expression using PET. Ischemic tissue uptake decreased, with normal-to-ischemic uptake ratios of 10.47 ± 3.03 (1.5 min) and 3.92 ± 1.12 (27.5 min) (*P* = 0.025). The reduction in [^18^F] FEDAC uptake in the PET image is consistent with the observed histopathological features. This suggests that this technique has good specificity and sensitivity in assessing the ischemic distribution, but more research should be needed to verify this technique.

However, the shortcomings of PET myocardial perfusion imaging in detecting coronary microvascular angina pectoris are that its spatial resolution is relatively low (58). The tracer used will inevitably cause radiation to patients. Compared with two-dimensional PET, the new high-sensitivity 3D positron emission tomography scanner adopts a three-dimensional acquisition method with a small radiation dose, which shows promising results in the experiment. It may diagnose CMVA more accurately (59).

### Cardiac Magnetic Resonance Imaging

Compared with radioactive tracer imaging, MRI has higher spatial resolution and temporal resolution (60), has the characteristics of no ionizing radiation, multi-parameter imaging, and good imaging of soft tissue, and is a powerful tool for clinical diagnosis of coronary artery diseases (61). After injection of contrast media, the signal intensity in areas with good coronary microcirculation increased uniformly, while in areas with myocardial ischemia, the signal intensity increased slowly. The MBF at rest and filling state can be obtained by analyzing the signal intensity by computer. Then CFR can be calculated. The research results of Raksha Indorkar (62) show that CFR derived from CMR is an independent predictor of major adverse cardiovascular events (MACE). CMR can observe coronary microcirculation in a non-quantitative or quantitative way. Quantitative CMR can accurately detect coronary microvascular dysfunction. This point has been confirmed by Zorach et al. (63). In addition, CMR is sensitive to early coronary microvascular lesions. Levelt et al. (64) performed adenosine stress and resting T1 mapping on 31 T2DM patients and 16 healthy controls. All patients were excluded from the obstructive coronary heart disease. They found that the myocardial perfusion reserve index (MPRI) of T2DM patients was lower than that of the control group (1.60 ± 0.44 vs. 2.01 ± 0.42, *P* = 0.008). During adenosine stress, patients with T2DM showed decreased non-contrast T1 response to relative stress vs. control group. So CMR can detect subclinical coronary microvascular dysfunction without gadolinium contrast agent injection. Studies suggest that CMR has a high application value in practice because the quantification of adenosine stress T1 CMR can accurately distinguish obstructive epicardial CAD from microvascular dysfunction without using contrast media (65).

Recently, in the diagnosis of microvascular diseases, Chatzantonis et al. (66, 67) introduced a new cardiovascular magnetic resonance (CMVD) parameter named “myocardial transit-time” (MyoTT), which is defined as the blood circulation time from the orfice of the coronary artery to the collection of the coronary sinus. MyoTT reflects the transmission time of gadolinium in myocardial microvascular. Therefore, it can be used for the diagnosis of CMVD. Compared with CFR, MyoTT has the advantages of not prolonging the examination time of patients, being easy to operate. It considers the heterogeneity of microvascular's functional lesions and can quickly evaluate the overall pathological changes of myocardial infarction microvasculature. However, due to the lack of research on MyoTT, it is necessary to evaluate its diagnostic performance in the future further.

Though little research has been done using perfusion CMR, recent studies have shown that MPRI derived from CMR, expressed as the hyperemic-to-resting myocardial perfusion ratio, can evaluate the prognosis of patients. In a retrospective study of 218 patients by Zhou et al. (68), 91% of the patients had an MPRI value of <2.0. During the 5.5-year follow-up, 34 MACEs occurred, an MPRI cut-off value of <1.47 was identified as the optimal prognostic threshold for predicting MACE.

Patients with cardiac electronic implant devices undergoing CMR may develop arrhythmia or even cardiac arrest. This is a major safety issue for CMR testing. Canadian Heart Rhythm Society and Canadian Association of Radiologists has detailed the precautions and taboos of CMR checks in the statement (69). With these electronic implantable devices being upgraded, their compatibility with CMR is also improved. As long as the manufacturer's agreement is strictly followed and supervision is done during the inspection, the inspection can be implemented. Another safety issue is that some patients are allergic to agents, but this can be solved by taking a skin test before injection or through drug replacement.

In addition, compared with 1.5T semi-quantitative CMR, 3T high-resolution CMR can identify CMVD with high precision. It shows better diagnosis performance in experiments (70) and improves the image signal-to-noise ratio (71). With the development of technology, high-resolution CMR will hopefully be widely used in the clinical diagnosis of CMVD in the future. At present, the disadvantage of magnetic resonance is the inability to provide examinations for patients with metal implants, high prices and long imaging time. So CMR equipment's development direction in the future is to solve the compatibility between metal implants and CMR, reduce the detection cost and time, and find simpler and feasible parameters to diagnose CMVD.

### Myocardial Contrast Echocardiography

The appearance of an acoustic contrast agent makes it possible to evaluate cardiac microcirculation by ultrasound. These contrast agents are composed of microbubbles similar to red blood cells. They can freely pass through the pulmonary circulation after entering the human body and enter the coronary microvasculature. In the myocardium with good coronary microvasculature, microbubbles can flow normally. Then imaging can be obtained through the scattering effect of microbubbles. However, images of abnormal myocardium are different from normal.

MCE uses microbubbles as contrast agents, so selecting suitable microfoam materials impacts diagnostic efficacy significantly. Xu et al. (72) designed platelet membrane-coated particles with a porous polylactic-co-glycolic acid (PLGA) core coated with a platelet membrane shell, platelet membrane can bind to myocardial ischemia-reperfusion areas. Injury areas of the mouse cardiac muscle were evaluated using ultrasound. They found a significantly increased signal strength in the risk area, a technique that may contribute to the diagnosis of early myocardial injury. Current ultrasonic microbubbles are used in various materials, but people mostly make improvements around the stability and toxicity of microbubbles, so it is necessary to find microbubble materials targeted to myocardial ischemia.

For a long time, the accuracy of CMVD detection by MCE has been controversial (73). In fact, its reliability and accuracy have been confirmed, and its diagnostic performance is comparable to that of positron emission tomography, but it lacks the approval of relevant regulatory authorities (74). Yang et al. (75) performed quantitative MCE of adenosine triphosphate (ATP) stress on 227 patients with chest pain and no obvious coronary artery stenosis (<50%) to evaluate CFR. They found that diabetic patients have a higher possibility of cardiovascular adverse events and considered that quantitative MCE of ATP stress could be used to evaluate coronary artery microcirculation. CFR obtained by ultrasound has assignable significance for the prediction of adverse events. Cortigiani et al. (76) administered Dipyridamole Stress Echocardiography in 1,130 patients (including 341 diabetic and 789 non-diabetic patients) and assessed their left anterior descending CFR. They found the annual event rate in the diabetic and non-diabetic patients was 9.3 and 5.1%, respectively. Patients with abnormal CFR significantly increased event rates over patients with normal CFR (*p* < 0.0001). This fully affirms the prognostic value of ultrasound in patients with diabetes.

Ultrasound may have a unique therapeutic function due to its biological effects. Moccetti et al. (77) found that the cavitation effect produced by ultrasound microbubbles can make myocardial microcirculation release ATP and increase microcirculation blood flow. In primates, blood flow increases up to double. Given the portability of the ultrasound device, it is possible to use it to control the area of myocardial infarction. In addition, we can use microbubbles to transport some drugs and promote their absorption. Acidic fibroblast growth factor (FGF1) can resist oxidative damage, induce endothelial smooth muscle cell proliferation and promote angiogenesis. Lei Zheng et al. (78) delivered FGF1 to the myocardium of diabetic rats by using FGF1-loaded nanoliposomes combined with ultrasonic targeted microbubble destruction. Cavitation and mechanical effect produced by microbubble destruction can promote drug absorption, and improve myocardial microcirculation. This achieves good results in preventing diabetic cardiomyopathy. The limitation of MCE in evaluating coronary microvascular function is that its accuracy is interfered with by many factors, such as the technical level of operators and patient's situation (79). In a word, MCE is a potential technology with advantages of low cost, simple operation, and no ionizing radiation, these makes it play a unique role in the preliminary screening of coronary microvascular angina pectoris.

### Coronary Computed Tomography Angiography

Coronary computed tomography angiography (CCTA) is a reliable method for clinical coronary stenosis (80). However, the diagnosis effect of coronary microvascular abnormality is not good (81). What is essential is that CT can implement the “one-stop-shop” imaging of INOCA (82). The vascular functional information provided by Dynamic CT-MPI is combined with the vascular anatomical information provided by CCTA, which is beneficial for detecting the function of microvessels and the identification of sizeable vascular obstruction. Yu et al. (83) also found that the MBF measured by CT-MPI was associated with the heart rate, when the heart rate increment is >20, the MBF will increase dramatically. Therefore, the effect of the heart rate also needs to be considered when referring to the MBF.

Intracavity attenuation gradient (TAG) is a new CT parameter, which is defined as the linear regression coefficient between the attenuation of intracavity contrast agent and axial distance (84). Recently, based on the attenuation gradient (TAG), Kojima et al. (85) put forward a new diagnostic index, the Dynamic TAG Index (DTI), which overcomes the disadvantage that TAG is easily affected by other non-ischemic factors. When there is coronary ischemia, DTI will obviously increase. So DTI increases the value of CCTA in diagnosing functional coronary diseases. The result shows that DTI combined with CCTA has good consistency with PET-MPI and shows comparable performance. However, there are few samples in this study, and it needs to be verified in a giant sample experiment. In addition, Alam et al. (86) found that the increased epicardial adipose tissue (EAT) measured by CT was significantly correlated with MFR. It was proposed that the cut-off value of EAT_thickness_ >5.6 mm was the best value for detecting abnormal coronary microvascular function. This may provide a new reference direction for the examination of coronary microvascular function.

Comparison of microvascular angina pectoris detected by various imaging methods (Tables 1, 2)

**Table 1:** Comparison and progress of various equipment for the diagnosis of myocardial ischemia (87).

**Device**	**Accuracy**	**Practicability**	**Developments**
SPECT	Common	Good	CZT camera improves imaging quality and reduce radiation The new algorithm SIFADS can improve image quality
PET	Good	Common	A new generation of tracer ^18^F-flurpiridaz is promising to be widely used in clinics [^18^F]FEDAC reflects the damage degree of cells
CMR	Good	Good	New parameter MyoTT increases the value of CMR 3T High Resolution CMR may be widely used in clinic
MCE	Bad	Common	Microbubbles can combine targeted drugs to improve microvascular function
CCTA	Common	Common	DTI and EAT_thickness_ increase the value of CCTA in the diagnosis of coronary microvascular disease

**Table 2:** Characteristics of various imaging methods for microvascular angina.

**Device**	**Advantages**	**Disadvantages**
SPECT	Low costs Good practicability	Low sensitivity and specificity Radiation exposure
PET	Accurate quantification of MBF Molecular-level research	High costs Radiation exposure Higher requirements for hospital
CMR	No radiation exposure Sensitive to subtle changes Assessment without contrast agents	High costs Limited by metal implants Time consuming
MCE	Low costs, Suitable for screening Biological effect	Bad reliability
CCTA	Assessment of epicardial vessels and microvascular	Insensitive to microvascular disease Radiation exposure

## Conclusion

The cardiovascular complications are prevalent in diabetic patients., And a large proportion of patients have vascular diseases in the early stage of diabetes. Rapid and accurate diagnosis of CMVA can help patients' treatment strategies. Imaging equipment such as SPECT, PET, CMR, and MCE have played an essential role in the non-invasive evaluation of CMVA. Non-invasive methods are more acceptable to patients than invasive methods and can reduce unnecessary waste of resources. Simultaneously, the use of cheap screening techniques also contributes to early intervention in asymptomatic patients. With the development of science and technology, the reliability of these methods will be continuously improved. The emergence of new technologies and new parameters will enable them to provide a better basis for the diagnosis of CMVA. Despite the relevant advantages of non-invasive techniques, we should recognize some limitations. Vasodilators used in non-invasive techniques only evaluate their own ability to dilate blood vessels. Also, obstructive CAD must be excluded before the diagnosis of CMVD. Given the high incidence of obstructive angina pectoris in diabetic patients, it is more realistic to choose appropriate imaging methods to distinguish obstructive angina pectoris from CMVD. It can also reduce the economic burden on patients. With the continuous development of medical imaging, we must look for high-sensitivity detection measures for early detection and intervention to reduce cardiovascular events in diabetic patients.

## Author Contributions

ZW and YQ conceived the topic and wrote the first draft. LL, GF, CS, and YC went through the manuscript and tables. All authors revised and approved of the final daft.

## Funding

This work was supported as follows: Jiangsu Provincial Health Commission Project (M2020016); the Open Project Program of Guangxi Key Laboratory of Centre of Diabetic Systems Medicine (GKLCDSM-20210101-02); Zhenjiang social development (SH2018030).

## Conflict of Interest

The authors declare that the research was conducted in the absence of any commercial or financial relationships that could be construed as a potential conflict of interest.

## Publisher's Note

All claims expressed in this article are solely those of the authors and do not necessarily represent those of their affiliated organizations, or those of the publisher, the editors and the reviewers. Any product that may be evaluated in this article, or claim that may be made by its manufacturer, is not guaranteed or endorsed by the publisher.
